# Diffusion-Plating Al_2_O_3_ Film for Friction and Corrosion Protection of Marine Sensors

**DOI:** 10.3390/mi16121344

**Published:** 2025-11-28

**Authors:** Yaoyao Liu, Longbo Li, Daling Wei, Kangwei Xu, Liangliang Liu, Long Li, Zhongzhen Wu

**Affiliations:** 1 National Key Laboratory of Marine Corrosion and Protection, Luoyang Ship Material Research Institute, Luoyang 471023, China; lyy_strive@126.com (Y.L.); llb725@126.com (L.L.); dalingwei725@163.com (D.W.); xukangwei1989@163.com (K.X.); 2 School of Advanced Materials, Peking University Shenzhen Graduate School, Shenzhen 518055, China; liull620@163.com

**Keywords:** Al_2_O_3_ film, friction and corrosion protection, marine sensor, tribological property

## Abstract

To extend the service life of sensors in seawater, this work prepared an integrated diffusion-plated Al_2_O_3_ film using high-power impulse magnetron sputtering (HiPIMS). The tribological properties of the Al_2_O_3_ film in a marine environment were tested using a tribometer. The morphology and evolution of the Al_2_O_3_ film before and after the friction tests were investigated by characterization techniques such as field emission scanning electron microscopy (FESEM). The results demonstrate that the Al_2_O_3_ film exhibits excellent tribological performance in the marine environment, significantly enhancing the wear resistance of the substrate material. Furthermore, with the protection of the Al_2_O_3_ film, the designed pressure sensor achieved high-sensitivity detection of minute operational forces underwater. When applied to a robotic gripper for manipulation tasks, the coated underwater sensor enabled accurate perception of subtle motion states of the grasped objects.

## 1. Introduction

Covering 71% of the Earth’s surface, the ocean holds abundant resources such as oil, gas, minerals, and diverse organisms, yet it remains underexploited [1,2,3,4,5]. Currently, most grippers used by underwater robots are rigid in structure, which can easily cause damage to valuable and fragile objects during grasping operations. Some soft robotic grippers, due to a lack of tactile perception, also fail to discern the properties of the objects they handle, leading to grasping failures [6,7,8,9,10]. Tactile sensors can measure minute pressure signals, which offers a promising solution to meet the demand for tactile perception in robotic underwater operations [11,12,13,14]. However, the marine environment can lead to failure of these sensors due to friction and corrosion after multiple grasping cycles. Therefore, there is an urgent need to develop coating materials suitable for the ocean environment to extend the service life of tactile sensors [15,16,17,18,19].

Seawater is a highly corrosive electrolyte, rich in chloride ions (Cl^−^) that aggressively attack and break down the passive oxide layers on metals like steel and titanium, leading to rapid pitting and uniform corrosion. Alumina is an extremely stable and inert ceramic. It is chemically resistant to a wide range of environments, including saline solutions. As a coating, it acts as a highly effective barrier layer, physically separating the underlying metal substrate from the seawater. This prevents the electrochemical reactions that cause corrosion from initiating.

For marine application, Al_2_O_3_ excels as a corrosion-resistant, inert, insulating, and hard barrier coating. Al_2_O_3_ films are regarded as the most promising solid lubricant materials for achieving superlubricity due to their superior properties, including high hardness, low friction coefficient, excellent wear resistance, and corrosion resistance [20,21,22,23,24]. Al_2_O_3_ coatings can enhance the anti-friction and anti-wear capabilities of mechanical components, prolong their service life, and thereby significantly reduce production costs. They are outperformed by other materials in specific properties like extreme hardness (DLC), CrN/TiN, SiO2, and crack tolerance (polymers), but their unique strength is their combination of properties with exceptional stability. DLC (Diamond-Like Carbon) exhibits extreme hardness and wear resistance, very low friction, and good chemical resistance. But it presents poor corrosion resistance in marine environments and develops micro-cracks due to brittleness. CrN/TiN shows vary high toughness and crack resistance, but it possesses a metallic character, providing less corrosion protection than a more insulating materials would. Polymers with low surface energy, low friction, is soft and poor abrasion resistance, can degrade under UV exposure, permeable to ions over time. SiO2 can have good chemical resistance and high transparency, but it has generally lower hardness than Al_2_O_3_, which can make it more susceptible to hydrofluoric acid and strong caustics.

Studies by Zhou et al. [25,26,27] demonstrated that silicon-doped Al_2_O_3_ films prepared on titanium alloy substrates exhibit a low friction coefficient and high corrosion resistance in seawater environments. Yang and colleagues investigated the influence of hydrogen content in Al_2_O_3_ films on their tribo-corrosion performance and discussed the friction and corrosion behaviors of Al_2_O_3_ films in seawater, as well as in alcoholic and acidic environments [28,29]. They concluded that the friction behavior of Al_2_O_3_ films in aqueous environments is influenced not only by their mechanical properties but also by environmental corrosion and antibacterial properties [30,31].

This paper primarily focuses on studying the tribological performance of Al_2_O_3_ films in seawater, as well as their compositional and structural evolution before and after friction tests, aiming to provide an essential experimental basis for their widespread application in marine environments. Based on the Al_2_O_3_ coating, we further developed an underwater tactile sensor capable of accurately measuring minute external contact forces in marine environments. Furthermore, the sensor’s ability to provide tactile perception information for robots working in underwater scenarios was validated by identifying the motion state of the grasped object.

## 2. Materials and Methods

### 2.1. Design and Fabrication of the Diffusion-Plated Al_2_O_3_ Film

**Figure 1:**
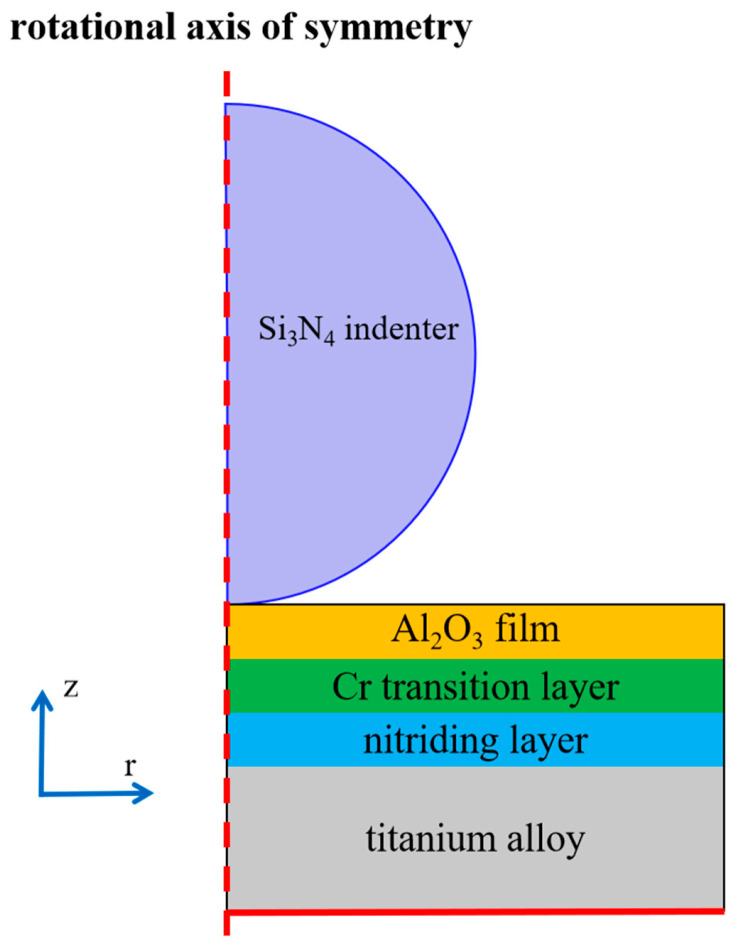
Schematic diagram of multi-layer stress behavior simulation model.

This study employs COMSOL Multiphysics 6.2 software to simulate the stress behavior of a multilayer structure under load. Given that the actual indenter has a spherical geometry, the indentation process into the multilayer structure is abstracted as a two-dimensional axisymmetric model. Consequently, the problem can be simplified into a revolving cross-section, as illustrated in Figure 1. The red dashed line represents the axis of rotational symmetry, along which the partial derivatives of all physical quantities with respect to the radial coordinate (r) are zero, satisfying the Riemann condition (i.e., the second type of boundary condition).The spherical indenter (purple), with a diameter of 5 mm, is made of Si_3_N_4_. Its hardness is significantly higher than that of the coating layers; thus, it is modeled as a rigid body without deformation. The multilayer structure, from bottom to top, consists of titanium alloy (gray), a nitrided layer (blue), a Cr interlayer (green), and an alumina thin film (orange). During the simulation of the indentation process, the bottom boundary of the multilayer structure (solid red line) is fixed, meaning its displacement is constrained to zero, satisfying the Dirichlet condition (i.e., the first type of boundary condition). The indenter is controlled to move downward under a constant load, allowing investigations into the stress distribution within the multilayer structure. This analysis aims to determine the stress state within each layer and at the interlayer interfaces.

**Figure 2:**
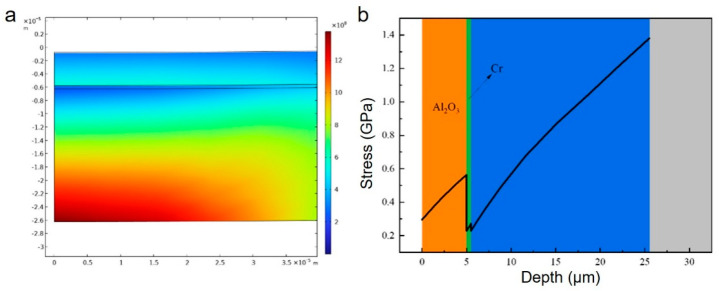
Stress distribution in the multi-layer structure of the alumina film. (**a**) Stress distribution map. (**b**) Two-dimensional stress distribution diagram.

Figure 2a illustrates the typical stress distribution in a multilayer structure (Al_2_O_3_ film thickness: 5 μm; AlN layer thickness: 20 μm; press-in depth: 1000 nm). The stress exhibits three distinct distribution regions corresponding to the Al_2_O_3_ film, Cr transition layer, and AlN layer. The stress variation trend along the symmetry axis (r = 0) is depicted in Figure 2b, revealing three continuous transition zones. Within each layer, stress progressively accumulates with increasing depth, reaching a maximum at the interface—a phenomenon prone to triggering the “thin ice effect”. Therefore, precisely controlling the thickness of each layer to confine the stress concentration within the layer itself rather than at the interface is crucial.

Given this, this work utilized the model to calculate stress state distributions under different loads (indentation depths of 30–1000 nm) and analyzed the effects of the nitrided layer’s thickness (10–200 μm), transition layer’s thickness (0.5–3 μm), and aluminum oxide film’s thickness (3–10 μm) on stress peak locations and distribution patterns. The model characterizes the magnitude of load on the indenter by penetration depth: deeper penetration indicates greater load, while shallower penetration indicates lesser load. We also measured the stress distribution at different indentation depths (Al_2_O_3_ thickness: 5 μm; transition layer thickness: 1 μm; nitrided layer thickness: 50 μm). Under low-load conditions (30 nm), the stress peak along the symmetry axis occurs between 2.5 and 3 μm, indicating that stress concentration primarily occurs within the Al_2_O_3_ film. As indentation depth increases, the stress concentration zone gradually shifts downward. At an indentation depth of 100 nm, the stress peak concentrates at the interface between the transition layer and the nitride layer. When the indentation depth exceeds 100 nm, the stress peak shifts into the nitrided layer. Notably, the stress extremum within the film (Al_2_O_3_ film and transition layer) remains at the interface between the Al_2_O_3_ film and transition layer. This indicates that under high loads, film delamination is more likely to be triggered, thereby inducing a “thin ice effect.” Therefore, to ensure the reinforced Ti alloy meets operational requirements, under low loads (indentation depth < 100 nm), the thickness of the Al_2_O_3_ film and nitrided layer should be controlled to confine the maximum stress within the Al_2_O_3_ film rather than at the interface, while under high loads (indentation depth exceeding 100 nm), the thickness of the nitrided layer must be controlled to ensure stress concentration occurs within the AlN layer itself, rather than within the Al_2_O_3_ film, in the transition layer, or at any interfaces.

**Figure 3:**
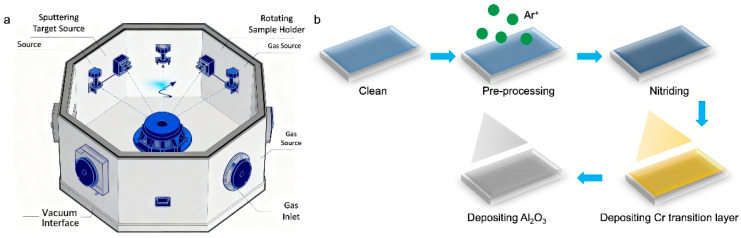
(**a**) Schematic diagram of the depositing equipment. (**b**) Fabrication process of the diffusion-plated Al_2_O_3_ film.

Based on the simulation results, diffusion-plating integrated technology was used to fabricate the composite Al_2_O_3_ film. The depositing equipment and fabrication process are shown in Figure 3. The fabricating process parameters employed were as follows:

1. Perform substrate nitriding using AEG at a nitriding pressure of 3.0 Pa, with an Ar flow rate of 80 sccm, a N_2_ flow rate of 120 sccm, a NH_3_ flow rate of 120 sccm, a nitriding bias voltage of 3000 V, and a nitriding time of 120 min.
2. Reduce the temperature to 100 °C and deposit a 1000 nm thick Cr transition layer via HiPIMS sputtering using a Cr target in an Ar atmosphere.
3. Employ HiPIMS to drive the Al target, depositing an Al_2_O_3_ layer under Ar and O_2_ atmospheres. Apply the following conditions: oxygen flow rate: 40 sccm; Ar flow rate: 50 sccm; frequency: medium (400 W, 400 V, 1 A); HIPIMS (2700 W, 700 V, 3.8 A, 50 Hz, 300 μs); deposition pressure: 0.3 Pa; ion source power: 1600 W; bias frequency: 20 Hz; pulse duration: 40 μs; pulse amplitude: −1600 V; deposition time: 60 min.
4. After cooling to room temperature, remove the sample.

To verify the reproducibility, three parallel deposition experiments were conducted under the optimized parameters. The deviations of key coating properties (e.g., thickness, hardness, and corrosion resistance) between parallel samples were less than 5%.

### 2.2. Characterization of the Al_2_O_3_ Properties

**Figure 4:**
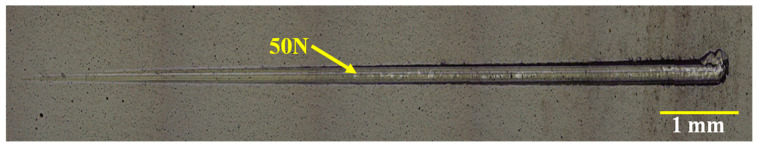
Morphology of the deposited alumina coating samples as determined by surface scratch test.

The final membrane layer properties were as follows:

The cross-section of the membrane layer revealed a dense sample structure, with an AlN layer depth of approximately 25 μm, a transition layer thickness of about 1 μm, and a surface Al_2_O_3_ layer thickness of approximately 3 μm. It was possible for the bond strength of the composite film layer to reach 50 N (Figure 4). We will explain the specific reasons for the high bonding strength of the composite film. Firstly, the transition layer is the most critical factor. Instead of a sharp, discrete boundary between the coating and the titanium substrate, diffusion plating creates a compositionally graded transition zone. In addition, the design of the intermediate layer allows for the mismatch between the structure, hardness and thermal expansion coefficient to be more gradual; the thermal stress is distributed gradually and a high concentration of stress at a single interface is avoided. Finally, the nitride layer is itself an excellent hard coating and has a high affinity for oxygen. When depositing Al_2_O_3_, we observed that it was able to easily form much stronger chemical bonds with oxygen or aluminum (such as Al-O-N or Al-N-O type bonds) than seen in the direct bonding of a metal substrate with Al_2_O_3_.

**Figure 5:**
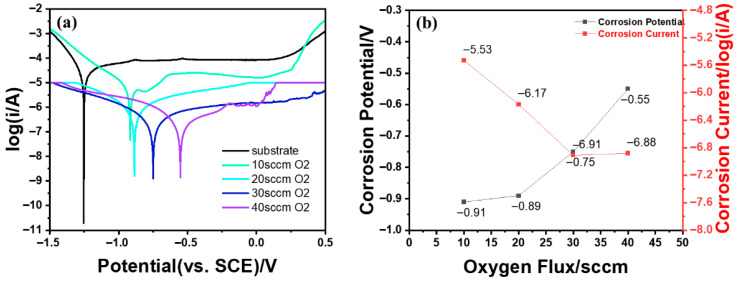
Preparation of alumina samples under different oxygen flow rates. (**a**) Tafel curve and (**b**) corrosion potential and corrosion current of the samples.

The corrosion potential, corrosion current, and other corrosion resistance properties of the alumina film were tested using a CHI 660E electrochemical workstation. The corrosion medium employed was a 3.5 wt% sodium chloride solution, with a Pt electrode servicing as the counter electrode and a saturated calomel electrode used as the reference electrode. Figure 5a shows the Tafel curves of the samples. The Tafel curves reveal that as the oxygen flow rate increases, the corrosion potential of the samples gradually shifts toward more positive values, while the corrosion current decreases progressively, indicating a gradual improvement in corrosion resistance. Calculations and analysis of the corrosion current and potential reveal that with increasing oxygen flow rate, the corrosion potential rose from −0.91 V to −0.44 V. The corrosion current decreased from −5.53 A/m^2^ to −6.88 A/m^2^, representing a reduction of nearly two orders of magnitude (Figure 5b). This demonstrates a substantial improvement in corrosion resistance.

## 3. Results

### 3.1. Tribological Properties of Al_2_O_3_ in Seawater

Figure 4 describes the variation in the friction coefficients of Ti80 metal, 316L stainless steel, and infiltrated composite aluminum oxide films in marine environments. Tribotests were performed using a ball-on-disk tribometer test system in a seawater environment.

**Figure 6:**
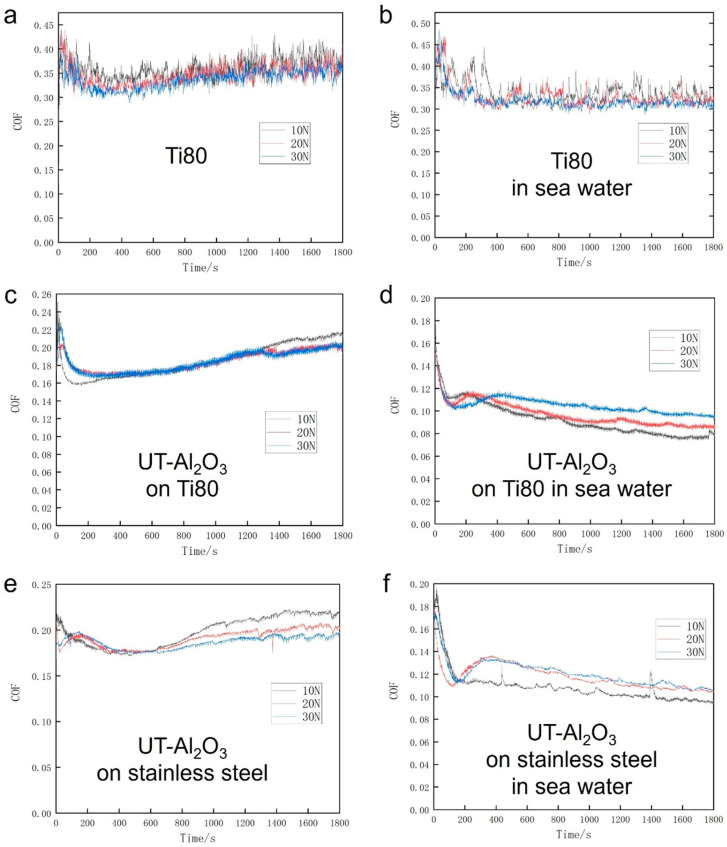
Tribological properties of Al_2_O_3_. (**a**) Friction coefficient of Ti80. (**b**) Friction coefficient of Ti80 in seawater. (**c**) Friction coefficient of Al_2_O_3_ on Ti80. (**d**) Friction coefficient of Al_2_O_3_ on Ti80 in seawater. (**e**) Friction coefficient of Al_2_O_3_ on stainless steel. (**f**) Friction coefficient of Al_2_O_3_ on stainless steel in seawater.

The friction coefficient results are shown in Figure 6. The dry friction coefficient of the Ti80 substrate was approximately 0.32. After depositing the aluminum oxide coating, the friction coefficient decreased to around 0.17. However, in the seawater environment, the friction coefficient further decreased to approximately 0.07. After depositing the aluminum oxide coating on the stainless-steel substrate, the coefficient of friction increased to approximately 0.2. However, in the seawater environment, the coefficient of friction decreased to around 0.09 and continued to show a downward trend. From the results, it can be concluded that the friction coefficients in a seawater environment were greatly reduced by Al_2_O_3_ film.

**Figure 7:**
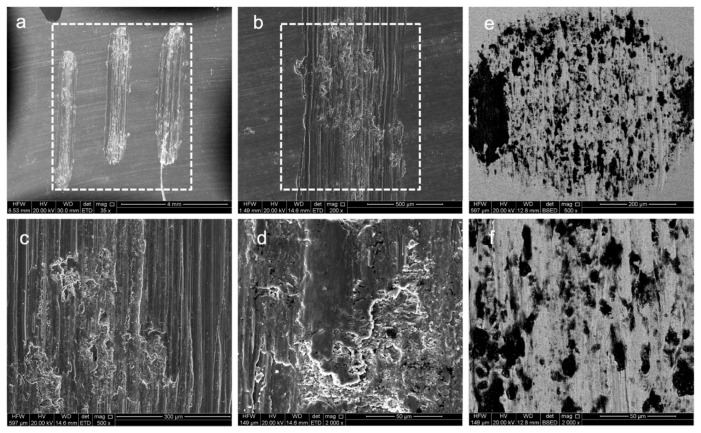
Analysis of Ti80’s surface morphology after friction. (**a**–**d**) Wear track of Ti80. (**e**,**f**) Wear scars from the sphere.

**Figure 8:**
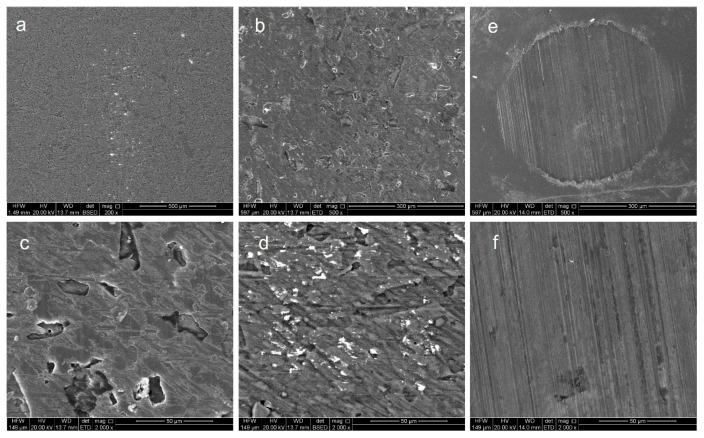
Analysis of the effect of Al_2_O_3_ film on Ti80’s surface morphology under friction. (**a**–**d**) Wear track of Al_2_O_3_ film; (**e**,**f**) wear scars from the sphere.

To reveal the mechanism of friction reduction, surface morphologies after wear in seawater are illustrated in Figure 7 and Figure 8. Micro-cracks and spalling pits appear on the wear track of Ti80 due to the low hardness. In addition, plenty of adhesive materials can be observed on the wear scar of untreated Ti80, while those on Al_2_O_3_ films are not obvious. The deposition of alumina film transforms the tribological behavior of titanium alloys in seawater by shifting the dominant mechanism from tribo-corrosion to nano-scale polishing and third-body lubrication by a soft corrosion product. Firstly, alumina is ceramic with chemical internes, which completely isolates the reactive titanium substrate from the seawater electrolyte, preventing the direct corrosion and adhesion of Ti. In addition, alumina has higher hardness compared with bare titanium; thus, it has a much lower tendency for atomic-scale adhesion, which is a primary source of high friction. From the microscopic morphology of the wear marks and spots in Figure 5, we can also observe that the titanium alloy exhibits distinct adhesive wear, whereas the aluminum oxide film does not. Finally, unlike TiO_2_, Al_2_O_3_ is highly stable in neutral and near-neutral pH environments. The formation of a lubricating aluminum hydroxide layer is a critical step for achieving a low and stable friction coefficient. Al_2_O_3_ + 3H_2_O = Al(OH)_3_. Al(OH)_3_ is a much softer material with a layered crystal structure that exhibits low shear strength. It forms a very thin, gel-like film on the wear track.

### 3.2. The Sensing Performance of Marine Sensors

**Figure 9:**
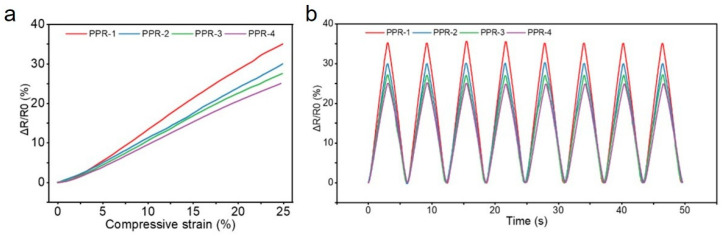
Compression strain responses of different structural frameworks. (**a**) Response of the sensor under 25% compression strain. (**b**) Response curve of the sensor under multiple cycles of compression.

Our underwater sensors are encapsulated with a metal casing. The coating is directly deposited onto the metal casing through HiPMS, thereby achieving wear resistance and corrosion protection for the sensors. The resistance response data of the sensors, obtained by applying the same 25% compressive strain to four different skeletal structures using a universal material testing machine, are shown in Figure 9. The sensor exhibited a stable response, with the maximum resistance change rate reaching up to 35%. Under identical compressive strain, the relative resistance change in the sensor gradually increased with the rise in the strain amplification factor of the skeletal structure, indicating the regulatory effect of the skeletal structure on the sensing sensitivity. The similar response curves and minimal peak drift during different loading cycles of compressive strain demonstrate the stability of the double-row convex skeletal structure under compression.

**Figure 10:**
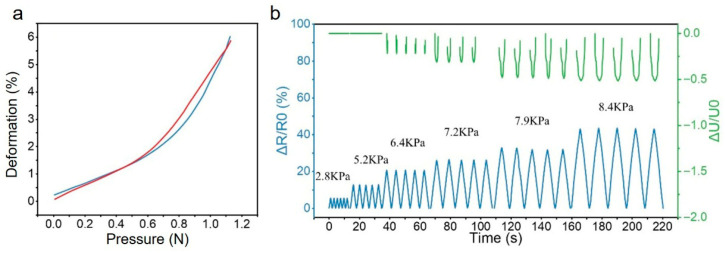
Sensor with an outwardly protruding frame structure sensing the stress signal. (**a**) Pressure–deformation curve of the sensor integrated with PPR-1 (Red line is the first test, blue line is the second test). (**b**) Responses of our sensor and a commercial piezoresistive sensor to different pressure stimuli.

Due to the dual sensitization effects of the strain concentration structure and the convex skeletal structure, the gauge factor (GF) of the sensor increased from an initial value of 0.31 to 1.3, representing a fourfold enhancement in perception sensitivity. Furthermore, the incorporated convex skeletal structure was able to convert external pressure signals into tensile strain signals within the sensor, thereby enabling the perception of pressure. The pressure sensing performance of a sensor varies with its stiffness. A sensor with lower stiffness can undergo significant deformation under minute pressure signals, resulting in a larger output sensing signal. In contrast, a sensor with higher stiffness exhibits minimal deformation under small pressure signals but can withstand larger pressure loads, characterized by a wide pressure sensing range but lower sensitivity to minor pressures. In this work, the convex skeletal structure influenced the overall equivalent stiffness of the prepared sensor. Stiffness tests conducted on a sensor with the skeletal structure designation PPR-1 using a universal material testing machine revealed that the sensor possessed low tensile/compressive stiffness, allowing it to generate substantial deformation under minimal pressure (Figure 10a). Subsequently, a commercial sensor (FSR402 resistive thin-film pressure sensor) was attached to the indenter of the universal testing machine. Both the prepared sensor and the commercial sensor were subjected to pressure signals simultaneously, and the comparative resistance responses are shown in Figure 10b. Owing to the low tensile/compressive stiffness of the overall structure, the prepared sensor exhibited a significant response even under small pressures, and its response gradually increased with further pressure application, demonstrating its accurate perception of both small and large pressure signals. The initial resistance of the commercial resistive thin-film pressure sensor was essentially infinite, decreasing to a normal range upon pressing. To facilitate signal acquisition and the calculation of relative changes, the commercial sensor was connected in series with a 40 kΩ fixed resistor across a 3.3 V voltage source, and the voltage change across the commercial sensor was measured as its response signal. Since small pressure signals did not reach the activation threshold of the commercial sensor, it showed no response at low strains. Moreover, even in the lower pressure phase of a larger pressure signal, the resistance of the commercial device remained largely unchanged, indicating its insensitivity to small pressure signals.

**Figure 11:**
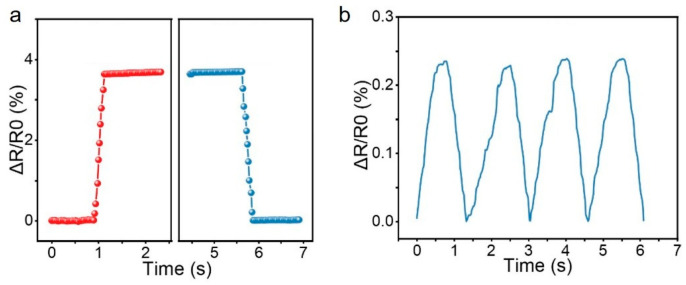
Sensor (**a**) response recovery time (Red and blue are response time and recovery time, respectively) and (**b**) minimum detection pressure.

Furthermore, the performance of the sensor with a double-row convex skeletal structure was characterized in terms of response time, cycling stability, and minimum pressure detection limit. First, the response time of the sensor was tested using a universal material testing machine. The indenter was controlled to descend at a speed of 50 mm/s with a compression displacement of 0.5 mm. The compression process was completed within 10 ms, while the sampling interval of the benchtop digital multimeter (DMM6500) was 20 ms. The time taken for the sensor’s data to stabilize after the compression strain was applied was recorded as the response time, and the time taken for the data to stabilize after the compression strain was released was recorded as the recovery time. As shown in Figure 11a, the response time of the sensor is 180 ms, and the recovery time is 200 ms.

On the other hand, the minimum pressure detection limit of the sensor was characterized. The indenter of the universal material testing machine was controlled to apply a pressure load at a slow speed of 1 mm/min. The loading time was set to 0.5 s, and the pressure applied to the sensor was 0.04 N. The resistance response of the sensor is shown in Figure 11b. When the loading time was further shortened and the pressure applied to the sensor was reduced, the resulting response curve of the sensor showed no distinguishable resistance change clearly attributable to the pressure application. Thus, the minimum pressure detection limit of the sensor was determined to be 0.04 N.

### 3.3. Applications of Marine Sensors

**Figure 12:**
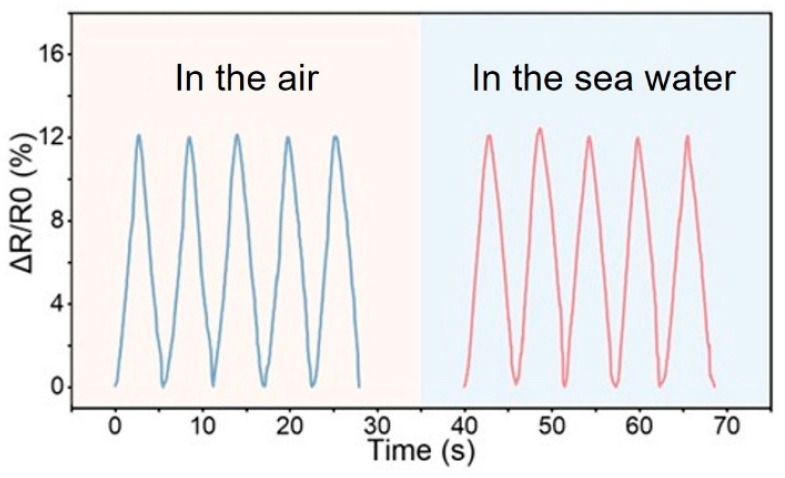
The response of the sensor to the same pressure signal in air and underwater.

Thanks to the excellent protective performance of the Al_2_O_3_ coating, our sensor can achieve highly sensitive perception of tiny forces in the marine environment without failure. For underwater biological sampling tasks, accurately perceiving the motion state of the organism concerned is crucial yet challenging. Failure to correctly identify the real-time motion state of the captured organism may lead to escape, death, or irreversible damage. Accurate identification of the organism’s state helps in adjusting the grasping strategy timely and keeping it alive while minimizing harm. Marine sensors, capable of detecting minute pressure signals with high accuracy, present a potential solution for precisely perceiving the motion state of grasped objects. To further validate the marine sensors’ accuracy of perception of the object’s motion state, a bionic fish model was used as the target object. A two-finger gripper (2FINGER-85) performed the grasping task, while the sensor measured the different motion states of the bionic fish during grasping. First, it was necessary to verify that the sensor’s perception performance underwater was consistent with its performance in air. The sensor was attached to the bottom of an evaporating dish, which was placed on the lower fixture of a universal material testing machine. A T-shaped acrylic block was clamped on the upper fixture of the machine to apply downward pressure on the sensor. The upper fixture was controlled to move downward at a specific speed, applying pressure signals to the sensor. The sensor’s response data during multiple pressing cycles in both air and water environments are shown in Figure 12. It can be observed that the sensor exhibits nearly identical response curves for the same pressure signal in both environments, indicating that underwater environments have minimal impact on the hydrogel sensor’s perception performance.

**Figure 13:**
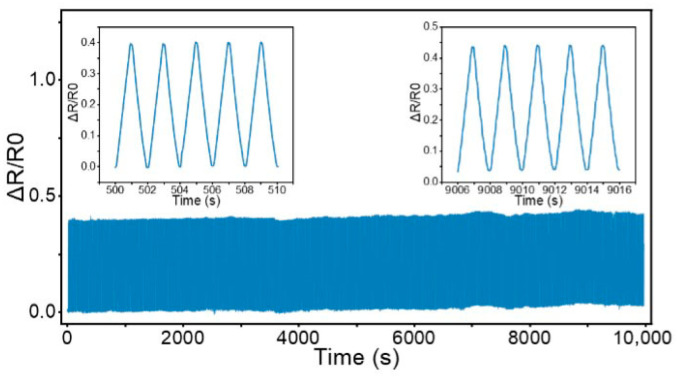
Results of tests on the underwater stability and durability of the sensor.

We conducted an underwater durability test for the sensors. From the experimental results, it can be seen that after 10,000 cycles, the sensor can still output stable electrical signals, indicating that the sensor has good stability and durability (Figure 13).

**Figure 14:**
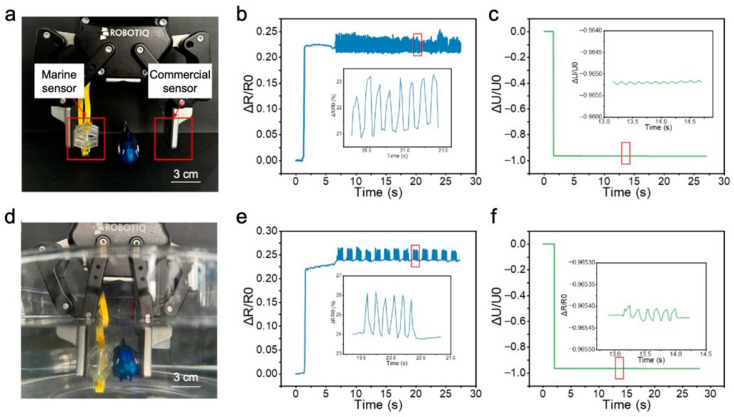
Perception of the motion state of the captured object. (**a**) The marine sensors and commercial sensors attached to the robotic hand. (**b**) The marine sensors perceiving the continuous swaying state of the bionic fish. (**c**) The marine sensors perceiving the continuous swaying state of the bionic fish. (**d**) The robotic hand placing the bionic fish in the water. (**e**) The marine sensors perceiving the intermittent swaying state of the bionic fish. (**f**) The commercial sensors perceiving the intermittent swaying state of the bionic fish.

Subsequently, verification was conducted to assess the sensor’s accurate perception of the minute vibrational states of objects during actual grasping tasks. As shown in Figure 14a, the sensor and a commercial sensor were attached to the inner sides of a two-finger gripper, with a bionic fish positioned between the gripper’s fingers. When the two-finger gripper closed, the top of the sensor’s skeletal structure contacted0 the bionic fish. Further reducing the gripper’s closure degree compressed the sensor’s skeletal structure, achieving grasping of the bionic fish. The two-finger gripper was controlled to move according to the following sequence: At T = 0 s, the gripper remained open, and the robotic arm was controlled to position the bionic fish between the two fingers, preparing for grasping. At T = 2 s, the gripper closed, gripping the bionic fish, which remained stationary. At T = 5 s, the robotic arm was controlled to move, enabling the gripper to lift the bionic fish and place it into a water-filled container. Subsequently, the gripper maintained a 50% closure state.

The resistance response data of the sensor during the aforementioned grasping task are shown in Figure 14b,e. At the 2 s mark, the two-finger gripper grasped the bionic fish. The compression of the skeletal structure stretched the sensing element, leading to an increase in the sensor’s resistance. Between 2 and 5 s, the bionic fish was lifted and was prepared to be placed into water. The stable sensor data during this phase indicated a stable grasping state and relative stillness between the fish and the gripper. At 5 s, when the fish was placed into water, the sensor’s data showed no abrupt change, suggesting that the transition between air and water had little impact on its resistance. After 5 s, the submerged bionic fish started swinging its tail at a certain frequency, changing its motion state from being relatively stationary to vibrating at a fixed frequency. This vibration caused the bionic fish to periodically compress the sensor’s skeletal structure, resulting in synchronous fluctuations in the resistance signal. Specifically, the bionic fish exhibited two vibration modes underwater: a continuous tail swimming mode with sustained oscillations at a fixed frequency, and an intermittent mode involving periods of tail swinging followed by rest, repeated cyclically at the same frequency. The sensor accurately discerned these different motion states. The commercial sensor’s responses under these states are shown in Figure 14c,f. While it also responded to the swinging motions, the amplitude of these responses was significantly smaller compared to the change caused by the initial grasp, making it unable to accurately and rapidly detect the minute motions of the grasped object.

## 4. Discussion

To address the low sensitivity of the sensor, a sensitivity-enhancing structure was designed to regulate its sensitivity. This structure is based on the influence of the double-row convex skeletal structure on the material’s Poisson’s ratio. Considering that a highly positive Poisson’s ratio can convert compressive strain into tensile strain and amplify it proportionally to the Poisson’s ratio, four convex skeletal structures with different parameters were designed as the sensor’s sensitivity-enhancing structures. The theoretical Poisson’s ratios of these four structures range from 1.9 to 2.58. After integrating the four structures with the hydrogel sensor featuring a strain concentration structure, finite element simulation analysis indicated that the maximum local strain amplification factor could reach 4.35 times that without the structure. Measurements of the resistance response of the sensors with the four structures revealed that the perception sensitivity decreases as Poisson’s ratio and the strain amplification factor of the structure decrease, demonstrating the regulatory effect of the structure on perception sensitivity. The gauge factor (GF) of the sensor integrated with the skeletal structure with the largest Poisson’s ratio is four times that of a sensor without any structure, which aligns with the local strain amplification factor obtained from the finite element analysis.

Due to its ability to perceive minute pressure signals and its minimal susceptibility to the underwater environment, the sensor provides an effective solution for underwater sensing. Its performance was tested in operational tasks such as identifying the motion state of grasped objects. For tiny pressure signals, the hydrogel sensor can detect pressure signals as low as 2.8 kPa, whereas the commercial device shows no response as the signal does not reach its activation threshold. The sensor accurately detects different vibration modes of the bionic fish with a significant response, while the commercial device exhibits a much smaller response. The excellent sensing performance of this sensor gives it broad application prospects in the fields of underwater robot perception and dexterous manipulation.

## 5. Conclusions

This work prepared a diffusion-plated Al_2_O_3_ film using high-power impulse magnetron sputtering (HiPIMS). The tribological properties of the Al_2_O_3_ film in a marine environment were tested using a tribometer, which demonstrated that the friction coefficients under seawater are greatly reduced by Al_2_O_3_ film. Based on the strain amplification characteristics of convex skeletal structures with a high positive Poisson’s ratio, a strain sensor featuring a double-row convex skeletal structure was designed and fabricated. Structurally, the double-row convex skeletal configuration amplifies the local strain experienced by the sensor, thereby enhancing its sensing sensitivity. The prepared sensor outperforms commercial piezoresistive sensors in detecting minute pressure signals. A robotic gripper integrated with the sensor enables accurate detection of the subtle vibrational states of grasped objects.

## Data Availability

The original contributions presented in this study are included in the article. Further inquiries can be directed to the corresponding authors.
